# The coverage rates for influenza vaccination and related factors in Korean adults aged 50 and older with chronic disease: based on 2016 Community Health Survey data

**DOI:** 10.4178/epih.e2018034

**Published:** 2018-07-24

**Authors:** Kyeong Hyang Byeon, Jaiyong Kim, Boyoung Choi, Bo Youl Choi

**Affiliations:** 1 Department of Public Health, Graduate School, Hanyang University, Seoul, Korea; 2 Department of Preventive Medicine, Hanyang University College of Medicine, Seoul, Korea; 3 Department of Public Health and Medical Administration, Dongyang University, Yeongju, Korea

**Keywords:** Influenza vaccination, Chronic disease, Adult, Republic of Korea

## Abstract

**OBJECTIVES:**

This study aims to identify the coverage rates for influenza vaccination and related factors depending on chronic disease in Korean adults aged 50 and older.

**METHODS:**

The 2016 Korea Community Health Survey was used for analysis. Chi-square test was performed to investigate the coverage rates for influenza vaccination depending on chronic disease, and a multiple logistic regression analysis was used to identify the factors associated with influenza vaccination, by chronic disease.

**RESULTS:**

In men with ≥1 chronic disease, 39.8% of 50-64 years of age, and 86.8% of elderly (over 65 years of age) received influenza vaccination. In women with ≥1 chronic disease, 58.7% of 50-64 years of age, and 89.9% of elderly (over 65 years of age) received influenza vaccination (p<0.001). The chronic diseases associated with influenza vaccination were hypertension (odds ratio [OR], 1.27; 95% confidence interval [CI], 1.19 to 1.37), diabetes (OR, 1.41; 95% CI, 1.28 to 1.55) in men aged 50-64, hypertension (OR, 1.34; 95% CI, 1.20 to 1.49), diabetes (OR, 1.17; 95% CI, 1.02 to 1.33), chronic cardiovascular disease (OR, 1.31; 95% CI, 1.07 to 1.60) in elderly (over 65 years of age). In women aged 50-64, hypertension (OR, 1.39; 95% CI, 1.30 to 1.49), diabetes (OR, 1.51; 95% CI, 1.35 to 1.68), chronic cardiovascular disease (OR, 1.31; 95% CI, 1.05 to 1.64), and hypertension (OR, 1.55; 95% CI, 1.40 to 1.71), diabetes (OR, 1.27; 95% CI, 1.12 to 1.43) in elderly (over 65 years of age).

**CONCLUSIONS:**

Populations in aged 50-64 are recommendation subject for vaccination or classified as high-risk group in case with chronic disease. Though subject over 60 years old is age close to the elderly, the coverage rates for vaccination was low. It is necessary to devise strategies to raise the coverage rates for vaccination.

## INTRODUCTION

Influenza is an acute respiratory disease caused by influenza virus infection. Elderly persons and patients with chronic diseases are defined as a high-risk group as they are at high adult, Korea Community Health Survey (KCHS), Korea risk of contracting influenza or complications. The high-risk group is at high-risk of complications such as pneumonia. Some patients require hospitalization and intensive care due to severe complications and die in some cases [1].

According to the Korea’s National Health Insurance Service data (2015), there were 5,293 patients aged 65 years or older, 5,453 patients aged 50-64 years, and 6,412 patients aged 30-40, who were seasonal influenza; the number of patients hospitalized for influenza increased as age decreased [2]. The number of inpatients per 100,000 registered residents was 78.1 for those aged 65 years or older, 48.7 for those aged 50-64 years, and 38.8 for those aged 30-49 years [3]. Since influenza can be prevented by prophylactic vaccination, receiving a vaccination before an outbreak occurs is essential, and an influenza outbreak can cause public health problems in local communities [4].

Vaccination is the most effective method to prevent influenza. The effect of vaccination varies by age and underlying disease. Vaccination is 70-90% effective in healthy adults, 50-60% effective for elderly patients who are hospitalized for influenza, and 80% in preventing death [5]. In chronic patients, vaccination can decrease the risks of influenza-like illnesses, pneumonia, hospitalization, and death [6]. In Korea, priority subjects for vaccination including those aged 65 years or older have been consistently instructed to receive vaccination even before an influenza outbreak alert is released, and those who have not been vaccinated are recommended to receive vaccination even if an outbreak has already occurred [7,8].

In the US, the coverage rates of vaccination was 63.4% for persons aged 65 years or older and 43.6% for those aged 50-60 years. In Korea, the rate of vaccination was 81.7% for those aged 65 years or older and 28.4% for those aged 50-64 years [5,9-11]. The rate of vaccination among those aged 50-64 years in Korea can be considered quite low considering that these subjects were the priority influenza vaccination group.

Healthy People 2020 of the US aims to raise the rate of influenza vaccination among elderly persons aged 65 years or older and patients with chronic diseases, who are classified as high-risk groups, to 90%. Korea aims to raise the vaccination rate among elderly persons aged 65 years or older to 82% but has not clarified the target vaccination rate for patients with chronic diseases who are classified as high-risk groups. Previous studies have reported low vaccination rates among patients with chronic diseases [12,13].

This study was conducted to investigate the rate of influenza vaccination in patients with chronic diseases who were aged 50-64 years in 2016 and to identify the factors that affect the actual vaccination rate to ultimately obtain basic research materials for increasing the vaccination rate among persons aged 50-64 years.

## MATERIALS AND METHODS

### Subjects and materials

The original data of the 2016 KCHS were used. 56,917 men and 73,693 women who responded to an influenza vaccination questionnaire were included [14].

### Variable selection and definition

In this study, the dependent variables were the variables of influenza vaccination. Persons who were vaccinated were defined as “yes”, and those who were not were defined as “no”.

The socioeconomic factors of the independent variables included gender, age, city and province, family income, education level, occupation type and marital status. The variables of health behaviors included smoking, drinking, walking, and subjective health status. Chronic diseases included hypertension, diabetes, myocardial infarction or angina, and stroke. Since all chronic diseases except for hypertension were associated with a high-risk of severe influenza or complications, patients with these chronic diseases were defined as high-risk groups [1]. For use of medical services, health examination, and use of health centers were used.

### Statistical analysis

The KCHS data are complex sample data. Weighted values, strata, and clusters were included in each stage of analysis and analyzed using a SURVEY procedure.

A chi-square test was performed to investigate significant differences in the socioeconomic factors, health behavior factors, chronic disease status, use of medical services, and influenza vaccination among the subjects. A multiple logistic regression analysis was performed to investigate the factors that affect the influenza vaccination coverage rate among subjects aged 50 years or older. Regions were divided into province, metropolis, medium cities and rural areas, and the coverage rate of influenza vaccination in subjects aged 50 years or older was investigated according to whether or not the subjects had at least one chronic disease.

Data were analyzed using SAS version 9.4 (SAS Institute Inc., Cary, NC, USA).

## RESULTS

### Influenza vaccination coverage rates by age among subjects aged 50 years or older

Vaccination coverage rates by age were 23.2% for men aged 50-54 years, 31.3% for women aged 50-54 years, 76.9% for men aged 65-69 years, and 83.3% for women aged 65-69 years (Table 1). The highest vaccination coverage rates were observed among men and women aged 60-64 years among all subjects aged 50-64 years. High influenza vaccination coverage rates of 90.2% were observed for men aged 80-84 years and of 91.1% for women aged 75-79 years among all subjects aged 65 years older.

### Health-related factors and characteristics of influenza vaccination coverage rates among subjects aged 50 years or older

Regarding vaccination coverage rates among subjects aged 50-64 years and those aged 65 years or older, the vaccination coverage rate was 30.5% for men aged 50-64 years, 43.8% for women aged 50-64 years, 84.3% for men aged 65 years or older, and 87.8% for women aged 65 years or older; women showed higher vaccination coverage rates (Table 2). In the low household income group, the vaccination coverage rate was 35.9% for men aged 50-64 years, 52.1% for women aged 50-64 years. Men aged 65 years or older had the highest vaccination coverage rate of 85.1%. There was no significant difference in the vaccination according to the household income among women aged 65 years or older. The vaccination decreased as the education level increased among men and women aged 50-64 years and women aged 65 years or older. There was no significant difference in the vaccination coverage rate according to the education level among men aged 65 years or older. Regarding vaccination coverage rates by occupation, vaccination coverage rates were higher among unemployed subjects in all age and gender groups. Regarding vaccination coverage rates by marital status, the vaccination coverage rate was 31.2% for men aged 50-64 years who had a spouse or cohabitant and was high at 85.1% for men aged 65-year or older who had a spouse or cohabitant. The vaccination coverage rate was 46.3% for women aged 50-65 years whose marital status was others (divorced, widowed, separated) and was the highest at 90.2% among single women aged 65 years or older. Higher vaccination coverage rates were observed for men who were former smokers and women who were non-smokers. The vaccination coverage rate was high at 88.3% for both men and women aged 50-64 years and women aged 65 years or older who did not drink alcohol. High vaccination coverage rates were observed among women aged 50-64 years and men and women aged 65 years older who engaged in walking activities. Higher vaccination coverage rates were observed for subjects with poorer subjective health statuses in all groups. Higher vaccination coverage rates were observed for both men and women who had health examinations and visited public health centers.

### Status of chronic disease and characteristics of influenza vaccination coverage rates among subjects aged 50 years or older

High vaccination coverage rates were observed among men and women with hypertension, diabetes, chronic cardiovascular disease or stroke. Significant difference in the vaccination coverage rate was observed among men and women aged 50-64 years according to whether or not they had stroke (Table 3). Regarding vaccination coverage rates for those with at least one chronic disease, the vaccination coverage rate was 39.8% for men aged 50-64 years, 58.7% for women aged 50-64 years, 86.8% for men aged 65 years or older, and 89.9% for women aged 64 years or older. Higher vaccination coverage rates were observed when the subjects had chronic diseases. Regarding vaccination coverage rates by the number of chronic diseases, the vaccination coverage rate was 39.3, 43.1 and 53.8% in the presence of one, two and three chronic diseases, respectively, among men aged 50-64 years. The vaccination coverage rate was 58.2, 65.3, and 88.7% in the presence of one, two and three chronic diseases, respectively, among women aged 50-64 years. The vaccination coverage rate was 86.3, 89.0, and 89.9% in the presence of one, two and three chronic diseases, respectively, for men aged 65 years or older, and 90.0, 90.0, and 83.6% in the presence of one, two and three chronic diseases, respectively, among women aged 65 years or older. Therefore, the vaccination coverage rate increased as the number of accompanying chronic diseases increased.

### Factors affecting influenza vaccination among subjects aged 50 years or older

Regarding the factors affecting influenza vaccination among men, the vaccination coverage rate was 19.0% lower for men aged 50-64 years in the middle household income group (95% confidence interval [CI], 0.70 to 0.92). The vaccination coverage rate was 29.0% lower for men aged 50-64 years who completed high school education than those who completed middle school education or lower (95% CI, 0.65 to 0.77) and was 43.0% lower for those who completed a university degree or above (95% CI, 0.52 to 0.63) (Table 4). Regarding vaccination coverage rates by occupations, the vaccination rate was 14.0% lower among men aged 50-64 years who did manual labor than office workers (95% CI, 0.78 to 0.94). The odds ratio (OR) for vaccination was high at 1.41 for men aged 65 years or over (95% CI, 1.13 to 1.17). The OR for vaccination was 1.23 times higher for single men aged 50-64 years relative to those who were married (95% CI, 1.02 to 1.47). For men aged 65 years or older, the OR for vaccination was 3.49 times higher (95% CI, 2.01 to 6.05) for those with a spouse and 2.57 times higher (95% CI, 1.47 to 4.50) for those with other marital statuses. Among men aged 50-64 years, the OR for vaccination was 1.48 times higher (95% CI, 1.38 to 1.60) for current smokers and 1.56 times higher (95% CI, 1.42 to 1.72) for non-smokers compared to former smokers. Among men aged 65 years or older, the OR was 1.61 times higher (95% CI, 1.43 to 1.81) for former smokers and 1.57 times higher (95% CI, 1.34 to 1.82) for non-smokers. Among men aged 50-64 years, the vaccination coverage rate was 19.0% lower for those who consumed alcohol (95% CI, 0.71 to 0.92). The OR for vaccination was 1.20 times higher (95% CI, 1.08 to 1.33) for men aged 65 years or older who engaged in walking activities. Among men aged 50-64 years, the vaccination coverage rate was 16.0% lower for those with poorer subjective health statuses (95% CI, 0.75 to 0.93). The vaccination coverage rate was 30.0% lower for men aged 65 years or older who had good subjective health statuses (95% CI, 0.61 to 0.81). The OR for vaccination was 2.29 times higher (95% CI, 2.09 to 2.51) and 2.68 times higher (95% CI, 2.39 to 3.00) for men aged 50-64 years and those aged 65 years or older who had health examinations, respectively. The OR for vaccination was 2.36 times higher (95% CI, 2.19 to 2.54) for men aged 50-64 years who visited public health centers and 4.31 times higher (95% CI, 3.85 to 4.82) for men aged 65 years or older who visited public health centers.

For women, the vaccination coverage rate was 14.0% lower for those aged 50-64 years with middle to high household incomes (95% CI, 0.77 to 0.95). The OR for vaccination was 1.40 times higher (95% CI, 1.14 to 1.70) for those aged 65 years or older. The vaccination coverage rate was 34.0% for women aged 50-64 years who completed high school education relative to those who completed middle school education or below (95% CI, 0.62 to 0.71). The vaccination coverage rate was 47.0% lower (95% CI, 0.48 to 0.58) for women who completed a university degree or above. The vaccination coverage rate was 13.0% lower (95% CI, 0.78 to 0.94) for women aged 50-64 years who did manual labor. The OR for vaccination was 1.17 times higher (95% CI, 1.04 to 1.31) for unemployed women aged 50-64 years and 1.70 times higher (95% CI, 1.12 to 2.60) for unemployed women aged 65 years or older. Among the subjects aged 50-64 years, the OR for vaccination was 1.31 times higher (95% CI, 1.01 to 1.70) for former smokers relative to current smokers, and 1.55 times higher (95% CI, 1.31 to 1.83) for non-smokers relative to current smokers. Among the subjects aged 65 years or older, the OR for vaccination was 1.61 times higher (95% CI, 1.12 to 2.32) and 1.73 times higher (95% CI, 1.29 to 2.33) for former smokers and non-smokers relative to current smokers, respectively. The vaccination coverage rate was 16.0% lower (95% CI, 0.78 to 0.90) for women aged 50-64 years who consumed alcohol and 11.0% lower (95% CI, 0.81 to 0.98) for women aged 65 years who consumed alcohol. The OR for vaccination was 1.15 times higher (95% CI, 1.08 to 1.22) for women aged 50-64 years who engaged in walking activities. Among the subjects aged 50-64 years, the vaccination coverage rate was 9.0% lower (95% CI, 0.84 to 0.98) for those with normal health statuses and 23.0% lower (95% CI, 0.70 to 0.84) for those who had good health statuses compared with those with poor health statuses. The vaccination coverage rate was 23.0% lower (95% CI, 0.67 to 0.90) for women aged 65 years or older who had good health statuses. The OR for vaccination was 2.07 times higher (95% CI, 1.91 to 2.25) for those aged 50-64 years and 2.36 times higher (95% CI, 2.13 to 2.60) for those aged 65 years or older who had health examinations. The OR for vaccination was 1.77 times higher for those aged 50-64 years (95% CI, 1.66 to 1.88) and 3.66 times higher (95% CI, 3.31 to 4.05) for those aged 65 years or older who visited public health centers.

Table 5 shows the factors associated with chronic diseases that affect influenza vaccination among subjects aged 50 years older. The OR was 1.27 times higher (95% CI, 1.19 to 1.37) in the presence of hypertension and 1.41 times higher (95% CI, 1.28 to 1.55) in the presence of diabetes among men aged 50-64 years. Among men aged 65 years or older, the OR for vaccination was 1.34 times higher (95% CI, 1.20 to 1.49) for those with hypertension, 1.17 times higher (95% CI, 1.02 to 1.33) for those with diabetes and 1.31 times higher (95% CI, 1.07 to 1.60) for those with chronic cardiovascular diseases. Among women aged 50-64 years, the OR for vaccination was 1.39 times higher (95% CI, 1.30 to 1.49) for those with hypertension, 1.51 times higher (95% CI, 1.35 to 1.68) for those with diabetes and 1.31 times higher (95% CI, 1.05 to 1.64) for those with chronic cardiovascular diseases. Among women aged 65 years older, the OR for vaccination was 1.55 times higher (95% CI, 1.40 to 1.71) times higher for those with hypertension and 1.27 times higher (95% CI, 1.12 to 1.43) for those with diabetes.

### Influenza vaccination coverage rate according to whether or not subjects aged 50 years or older have at least one chronic disease in each region

Regarding influenza vaccination coverage rates by cities and provinces, South Jeolla Province had the highest vaccination coverage rate of 44.1%, and Jeju Island had the lowest vaccination coverage rate of 31.4% for those aged 50-64 years (Table 6). Incheon Metropolitan City had the highest vaccination coverage rate of 54.3%, and Jeju Island had the lowest vaccination coverage rate of 36.3% for those with at least one chronic disease. North Chungcheong Province had the highest vaccination coverage rate of 89.5%, and Gwangju Metropolitan City had the lowest vaccination coverage rate of 81.4% for those aged 65 years or older. Sejong Metropolitan Autonomous City had the highest vaccination coverage rate of 92.5% followed by 91.5% in North Chungcheong Province, and South Gyeongsang Province had the lowest vaccination coverage rate of 84.2% for those with at least one chronic disease.

**Figure 1.**
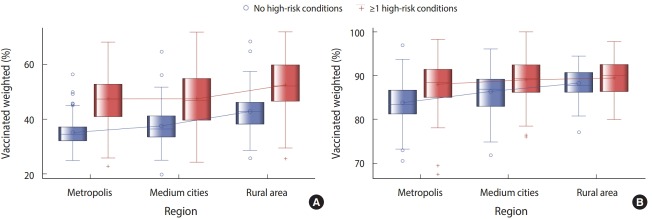
Coverage rates of influenza vaccinations by region in patients aged (A) 50-64 and (B) ≥ 65 years.

Regarding the number of metropolis, medium cities, and rural areass that did not reach the target vaccination coverage rate of 80.0% set in 2015 for persons aged 65 years or older, 16 metropolis, 6 medium cities, and 1 rural area did not reach the target vaccination of 80.0% for persons without any chronic diseases. Five metropolis, 4 medium cities did not reach the target rate for those with at least one chronic disease (Figure 1).

## DISCUSSION

This study used the 2016 KCHS data to investigate influenza vaccination coverage rates and associated factors among subjects aged 50 years or older who responded to a questionnaire about influenza vaccination history in the last one year. The influenza vaccination coverage rates were investigated by regions and accompanying chronic diseases.

In Korea, the scope of the National Immunization Program was expanded to civilian medical facilities in 2015, and its goal was set to reaching influenza vaccination coverage rates of 80.0% or higher for elderly persons aged 65 years or older [15]. The National Immunization Program provides free vaccination for persons aged 65 years or older and has helped reach high vaccination coverage rates of 80.0% or above. However, the vaccination coverage rate among men aged 65-69 years has been found to be 76.9%. Although persons aged 50-64 years were included in the priority vaccination group in 2003, vaccination coverage rates among men and women in this age group were found to be low at 30.5 and 43.8%, respectively. Therefore, policies and vaccination advertisements within communities are needed to increase vaccination coverage rates.

In Korea, vaccination is recommended for priority vaccination groups before an influenza outbreak occurs. Medical staff, pregnant women, persons aged 50-64 years, elderly persons aged 65 years or older, and patients with chronic respiratory diseases, chronic heart diseases, chronic liver disease, chronic kidney disease, nerve-muscle disease, hemato-oncologic disease or diabetes are included in the priority group. Of these, those with chronic diseases are classified as high-risk groups.

In this study, high vaccination coverage rates were found among subjects aged 50 years who had chronic diseases compared with those who did not. Consistent with overseas and domestic research findings, vaccination coverage rates increased as the number of chronic diseases increased among subjects with at least one chronic disease [16,17]. In addition, although persons aged 50-64 years who have chronic diseases and are thus classified as high-risk groups, or persons aged 65 years or older or persons with chronic diseases are at high-risk of severe influenza or complications, low vaccination coverage rates were found among subjects aged 50-64 years with chronic diseases. Since patients with chronic diseases have increased sensitivity to influenza infection, they are at higher risk for complications such as worsening of underlying diseases, pneumonia and impairment of other organs; as a result, their chronic diseases may become severe, or the patients may die. For this reason, influenza vaccination is important for these patient groups. Men aged 50 years or older with cerebrovascular diseases and women aged 50 years or older with diabetes showed high vaccination coverage rates. For both men and women, diabetes was identified as a factor that increased vaccination coverage rates. It appears vaccination coverage rates were higher among diabetic patients since these patients have reduced immunity against infections due to hyperglycemia and thus have serious accompanying complications such as pneumonia and septicemia.

Regarding the health-related factors, high vaccination coverage rates were observed for both unemployed men and women, and the unemployment status was identified as a factor that increased vaccination coverage rates among subjects aged 65 years or older. In a previous study, being too busy for vaccination was one of the reasons for being unable to receive vaccination [18,19]. It is possible that the unemployed subjects had higher vaccination coverage rates since they could afford more time for vaccination. Regarding the marital status, higher vaccination coverage rates were found for married men and women, and the ‘married’ marital status was identified as a factor that increased vaccination coverage rate among men. A domestic study reported high vaccination coverage rates among those who lived with a spouse or a family and explained that social networks and vaccination recommendations by family members positively affect health information [6,20]. Except for men aged 65 years who smoked and consumed alcohol, which are bad life habits, vaccination coverage rates were low for both men and women. Consistent with a previous study, it appears that persons with healthy lifestyles tend to seek healthy behaviors and preventive treatments and are highly interested in influenza vaccination [13]. Both men and women who had health examinations and visited public health centers showed high vaccination coverage rates. Both having health examinations and visiting public health centers were identified as the factors that increased vaccination coverage rates. Of the factors related to the use of medical services, contact with medical staff during a treatment was found to positively affect vaccination [17,21]. It appears that people who undergo health examinations and visit public health centers show greater interest in the early prevention of diseases and health-promoting behaviors and thus show higher vaccination coverage rates.

Regarding vaccination coverage rates by regions, high vaccination coverage rates were observed in metropolitan cities than provinces. It appears that cities show higher vaccination coverage rates since they allow easier access to medical institutions. It also appears that the expansion of the free vaccination services for persons aged 65 years or older to private medical institutions has increased vaccination coverage rates for those living in cities. Subjects living in metropolis, medium cities and rural areas who had at least one chronic disease showed high vaccination coverage rates. In all counties, the target vaccination coverage rate of 80.0% set in 2015 was reached for subjects aged 65 years or older with at least one chronic disease. The high vaccination coverage rates observed in counties were consistent with previous findings [22]. Vaccination advertisements and education have greater ripple effects in rural areas than cities since they are provided through community health centers in rural areas [23].

This study had a number of limitations. First, the influenza vaccination status was investigated using a self-report questionnaire. Therefore, the investigated vaccination coverage rates may differ from the actual vaccination coverage rates, and they may be overestimated. Second, the possibility of recall bias that may have occurred when the subjects were asked about their influenza vaccination status in the last one year in the KCHS cannot be eliminated. Third, due to the limitations of the questionnaire, vaccination coverage rates for various chronic diseases with high-risk of influenza complications could not be investigated. Fourth, although health professionals’ recommendations and previous history of vaccination can significantly affect influenza vaccination among elderly persons and patients with chronic diseases, these variables could not be investigated as they were not included in the KCHS data [24].

Despite these limitations, this study is meaningful in that it used survey data that considered regional characteristics to divide regions into metropolis, medium cities and rural areas and further divided the subjects into 50-64 and ≥65 years age groups to investigate vaccination coverage rates according to the chronic disease status and identify the factors that affect vaccination. The higher vaccination coverage rates observed for subjects aged 50-64 years who had at least one chronic disease compared to those who did not, and for subjects who lived in rural area than those who lived in medium cities indicate that active vaccination advertisements are needed to increase vaccination coverage rates among those aged 50-64 years who live in metropolis and medium cities. In addition, healthy professionals must recommend patients with chronic diseases who are aged 50-64 years to receive vaccination when they visit a health institution for a treatment.

## Figures and Tables

**Table 1.** Coverage rates of influenza vaccinations by age group in the study population

Age (yr)	Men		Women	
	Total	Vaccinated^***^	Total	Vaccinated^***^
50-54	10,018	2,359 (23.2)	11,300	3,841(31.3)
55-59	11,137	3,550 (29.8)	13,370	6,332 (44.6)
60-64	9,462	4,430 (43.1)	11,189	6,998 (59.3)
65-69	8,266	6,551 (76.9)	9,841	8,401(83.3)
70-74	7,195	6,327 (86.7)	9,818	8,933 (90.4)
75-79	6,193	5,591 (90.1)	9,037	8,290 (91.1)
80-84	3,373	3,054 (90.2)	6,062	5,443 (88.8)
≥85	1,273	1,088 (83.8)	3,076	2,584 (84.0)

Values are presented as number or number (weighted %).

*** p<0.001 by chi-square test.

**Table 2.** Coverage rates of influenza vaccinations by health-related factors in the study population

Variables	Men						Women					
	50-64 yr		p-value^1^	≥65 yr		p-value^1^	50-64 yr		p-value^1^	≥65 yr		p-value^1^
	Total	Vaccinated		Total	Vaccinated		Total	Vaccinated		Total	Vaccinated	
Total	30,617	10,339 (30.5)		26,300	22,611 (84.3)	<0.001	35,859	17,171 (43.8)		37,834	33,651 (87.8)	<0.001
Family income												
Low	3,263	1,284 (35.9)	<0.001	11,795	10,289 (85.1)	0.002	5,334	3,046 (52.1)	<0.001	22,135	19,816 (88.2)	0.14
Moderate-low	11,587	4,184 (32.4)		10,500	8,996 (85.0)		15,205	7,686 (47.2)		10,654	9,418 (87.6)	
Moderate-high	9,230	2,795 (28.0)		2,571	2,141 (81.5)		9,117	3,884 (40.2)		3,165	2,760 (86.4)	
High	6,172	1,977 (30.3)		1,247	1,038 (82.6)		5,790	2,382 (39.4)		1,608	1,424 (88.2)	
Education level												
≤ Middle school	9,322	3,952 (38.9)	<0.001	17,179	14,931 (85.0)	0.06	17,703	10,060 (53.9)	<0.001	34,238	30,591 (88.2)	<0.001
High school	12,209	3,845 (29.5)		5,927	5,021 (83.6)		12,746	5,240 (39.6)		2,604	2,245 (86.2)	
≥ College	8,998	2,517 (26.7)		3,158	2,633 (83.3)		5,315	1,824 (33.0)		953	782 (83.4)	
Occupation												
White-collar	6,497	1,951 (28.9)	<0.001	846	668 (79.5)	<0.001	3,191	1,178 (34.8)	<0.001	200	154 (79.5)	0.003
Blue-collar	16,562	5,559 (29.4)		9,692	8,420 (82.7)		14,401	6,864 (41.0)		8,322	7,499 (87.6)	
Unemployed	7,464	2,788 (34.6)		15,749	13,512 (85.1)		18,231	9,114 (47.1)		29,292	25,981 (87.9)	
Marital status												
Single	1,127	324 (27.8)	<0.001	101	70 (64.1)	<0.001	484	190 (36.8)	<0.001	148	128 (90.2)	<0.001
Married	26,185	9,068 (31.2)		22,941	19,891 (85.1)		28,606	13,629 (43.3)		17,142	15,419 (88.7)	
Other	3,259	938 (25.7)		3,234	2,634 (79.6)		6,721	3,339 (46.3)		20,527	18,090 (86.9)	
Smoking status												
Current smoker	11,735	3,180 (24.4)	<0.001	5,207	4,124 (76.7)	<0.001	1,112	437 (36.0)	<0.001	785	647 (80.0)	<0.001
Former smoker	13,425	5,117 (34.3)		15,634	13,785 (86.4)		647	299 (41.6)		1,157	994 (86.3)	
Non-smoker	5,457	2,042 (34.2)		5,456	4,699 (85.4)		34,100	16,435 (44.1)		35,891	32,009 (88.0)	
Drinking												
No	1,988	767 (35.3)	<0.001	3,470	2,949 (83.8)	0.52	9,207	4,815 (48.9)	<0.001	18,870	16,817 (88.3)	0.03
Yes	28,629	9,572 (30.3)		22,830	19,662 (84.4)		26,647	12,353 (42.3)		18,955	16,826 (87.3)	
Walking activity												
No	18,185	6,118 (30.3)	0.52	15,592	13,334 (83.2)	<0.001	21,651	10,173 (42.2)	<0.001	26,207	23,219 (87.4)	0.06
Yes	12,409	4,208 (30.8)		10,656	9,232 (85.5)		14,183	6,982 (45.8)		11,551	10,368 (88.4)	
Perceived health status												
Bad	4,807	1,984 (37.9)	<0.001	9,522	8,333 (86.1)	<0.001	7,223	4,111 (52.7)	<0.001	20,057	17,955 (88.7)	<0.001
Average	13,524	4,586 (30.7)		10,059	8,728 (85.3)		18,033	8,703 (44.5)		12,443	11,083 (88.2)	
Good	12,281	3,767 (27.9)		6,716	5,547 (80.8)		10,598	4,356 (37.4)		5,327	4,608 (84.0)	
Health screening												
No	6,290	1,243 (17.6)	<0.001	4,772	3,361 (70.1)	<0.001	5,906	1,883 (29.5)	<0.001	8,364	6,574 (78.7)	<0.001
Yes	24,317	9,095 (33.7)		21,508	19,235 (87.9)		29,945	15,282 (46.7)		29,417	27,032 (90.6)	
Public health centers use												
No	22,315	5,863 (26.8)	<0.001	8,949	6,433 (74.8)	<0.001	22,385	8,727 (39.6)	<0.001	11,812	9,164 (80.4)	<0.001
Yes	8,299	4,476 (48.8)		17,347	16,175 (93.0)		13,470	8,442 (55.3)		26,012	24,478 (94.0)	

Values are presented as number or number (weighted %).

1 Chi-square test.

**Table 3.** Coverage rates of influenza vaccinations by chronic disease status in the study population

Disease	Men						Women					
	50-64 yr		p-value^1^	≥65 yr		p-value^1^	50-64 yr		p-value^1^	≥65 yr		p-value^1^
	Total	Vaccinated		Total	Vaccinated		Total	Vaccinated		Total	Vaccinated	
Hypertension^2^												
No	21,080	6,513 (27.7)	<0.001	13,308	11,092 (81.2)	<0.001	26,192	11,598 (40.4)	<0.001	16,022	13,812 (84.5)	<0.001
Yes	9,533	3,824 (37.0)		12,987	11,515 (87.3)		9,665	5,573 (53.9)		21,803	19,833 (90.2)	
Diabetes												
No	26,119	8,392 (29.0)	<0.001	20,495	17,488 (83.5)	<0.001	32,356	15,000 (42.3)	<0.001	29,997	26,546 (87.1)	<0.001
Yes	4,495	1,947 (40.0)		5,800	5,120 (86.7)		3,498	2,171 (59.6)		7,827	7,098 (90.2)	
Chronic cardiovascular disease												
No	29,496	9,844 (30.2)	<0.001	23,832	20,382 (83.8)	<0.001	35,106	16,710 (43.5)	<0.001	34,950	31,046 (87.6)	0.04
Yes	1,114	494 (40.0)		2,443	2,210 (88.9)		742	457 (59.3)		2,807	2,545(89.5)	
Stroke												
No	29,969	10,053 (30.3)	<0.001	24,444	21,019 (84.2)	0.09	35,431	16,918 (43.6)	<0.001	35,824	31,896 (87.7)	0.34
Yes	646	285 (42.0)		1,846	1,584 (86.1)		424	251 (55.7)		1,997	1,744 (88.6)	
High-risk conditions^3^												
No	24,953	7,899 (28.6)	<0.001	17,773	15,094 (83.0)	<0.001	31,496	14,498 (42.0)	<0.001	26,762	23,666 (86.8)	<0.001
≥1	5,663	2,440 (39.8)		8,526	7,517 (86.8)		4,363	2,673 (58.7)		11,072	9,985 (89.9)	
High-risk conditions^4^												
No	24,953	7,899 (28.6)	<0.001	17,773	15,094 (83.0)	<0.001	31,496	14,498 (42.0)	<0.001	26,762	23,666 (86.8)	<0.001
1	5,099	2,169 (39.3)		7,093	6,241 (86.3)		4,071	2,474 (58.2)		9,608	8,664 (90.0)	
2	536	256 (43.1)		1,303	1,155 (89.0)		283	192 (65.3)		1,369	1,240 (90.0)	
3	28	15 (53.8)		130	121 (89.9)		9	7 (88.7)		95	81 (83.6)	

Values are presented as number or number (weighted %).

1 Determined using the chi-square test.

2 Excluded from the high-risk group.

3 Adults with one or more of the following: diabetes, myocardial infarction or angina, stroke.

4 Adults who had at least one of the following: diabetes, myocardial infarction or angina, stroke.

**Table 4.** Factors associated with Influenza vaccination in the study population^1^

Variables	Men				Women			
	50-64 yr		≥65 yr		50-64 yr		≥65 yr	
	Crude OR (95% CI)	Adjusted OR (95% CI)^1^	Crude OR (95% CI)	Adjusted OR (95% CI)^1^	Crude OR (95% CI)	Adjusted OR (95% CI)^1^	Crude OR (95% CI)	Adjusted OR (95% CI)^1^
Family income								
Low	1.00 (reference)	1.00 (reference)	1.00 (reference)	1.00 (reference)	1.00 (reference)	1.00 (reference)	1.00 (reference)	1.00 (reference)
Moderate-low	0.86 (0.77, 0.96)	0.90 (0.80, 1.03)	0.99 (0.89, 1.10)	1.02 (0.90, 1.15)	0.82 (0.75, 0.89)	096 (0.87, 1.05)	0.94 (0.85, 1.04)	1.04 (0.93, 1.16)
Moderate-high	0.69 (0.62, 0.78)	0.81 (0.70, 0.92)	0.77 (0.67, 0.90)	0.86 (0.73, 1.02)	0.62 (0.56, 0.67)	0.86 (0.77, 0.95)	0.85 (0.73, 0.98)	1.02 (0.87, 1.20)
High	0.78 (0.69, 0.88)	0.95 (0.82, 1.10)	0.83 (0.69, 1.01)	1.00 (0.80, 1.23)	0.60 (0.54, 0.66)	0.95 (0.84, 1.06)	1.00 (0.83, 1.22)	1.40 (1.14, 1.70)
Education level								
≤ Middle school	1.00 (reference)	1.00 (reference)	1.00 (reference)	1.00 (reference)	1.00 (reference)	1.00 (reference)	1.00 (reference)	1.00 (reference)
High school	0.66 (0.61, 0.71)	0.71 (0.65, 0.77)	0.90 (0.81, 1.00)	0.94 (0.83, 1.06)	0.56 (0.53, 0.60)	0.66 (0.62, 0.71)	0.83 (0.72, 0.96)	0.95 (0.81, 1.11)
≥ College	0.57 (0.53, 0.62)	0.57 (0.52, 0.63)	0.88 (0.77, 100)	0.95 (0.81, 1.11)	0.42 (0.39, 0.46)	0.53 (0.48, 0.58)	0.67 (0.54, 0.84)	0.79 (0.62, 1.00)
Occupation								
White-collar	1.00 (reference)	1.00 (reference)	1.00 (reference)	1.00 (reference)	1.00 (reference)	1.00 (reference)	1.00 (reference)	1.00 (reference)
Blue-collar	1.03 (0.95, 1.11)	0.86 (0.78, 0.94)	1.23 (1.00, 1.51)	0.94 (0.74, 1.19)	1.30 (1.18, 1.45)	0.87 (0.78, 0.98)	1.83 (1.23, 2.73)	1.21 (0.79, 1.87)
Unemployed	1.31 (1.20, 1.42)	1.05 (0.95, 1.17)	1.48 (1.22, 1.79)	1.41 (1.13, 1.77)	1.67 (1.52, 1.85)	1.17 (1.04, 1.31)	1.87 (1.27, 2.76)	1.70 (1.12, 2.60)
Marital status								
Single	1.00 (reference)	1.00 (reference)	1.00 (reference)	1.00 (reference)	1.00 (reference)	1.00 (reference)	1.00 (reference)	1.00 (reference)
Married	1.18 (0.99, 1.40)	1.23 (1.02, 1.47)	3.20 (2.00, 5.14)	3.49 (2.01, 6.05)	1.31 (1.03, 1.67)	1.04 (0.81, 1.34)	0.85 (0.49, 1.48)	0.60 (0.32, 1.11)
Other	0.90 (0.74, 1.09)	0.95 (0.77, 1.16)	2.18 (1.34, 3.54)	2.57 (1.47, 4.50)	1.48 (1.16, 1.89)	1.07 (0.83, 1.38)	0.72 (0.41, 1.25)	0.55 (0.30, 1.01)
Smoking status								
Current smoker	1.00 (reference)	1.00 (reference)	1.00 (reference)	1.00 (reference)	1.00 (reference)	1.00 (reference)	1.00 (reference)	1.00 (reference)
Former smoker	1.61 (1.51, 1.73)	1.48 (1.38, 1.60)	1.93 (1.72, 2.15)	1.61 (1.43, 1.81)	1.26 (0.99, 1.61)	1.31 (1.01, 1.70)	1.57 (1.12, 2.21)	1.61 (1.12, 2.32)
Non-smoker	1.61(1.47, 1.75)	1.56 (1.42, 1.72)	1.77 (1.54, 2.04)	1.57 (1.34, 1.82)	1.40 (1.20, 1.63)	1.55 (1.31, 1.83)	1.83 (1.40, 2.39)	1.73 (1.29, 2.33)
Drinking								
No	1.00 (reference)	1.00 (reference)	1.00 (reference)	1.00 (reference)	1.00 (reference)	1.00 (reference)	1.00 (reference)	1.00 (reference)
Yes	0.80 (0.70, 0.90)	0.81 (0.71, 0.92)	1.05 (0.91, 1.20)	0.96 (0.81, 1.12)	0.77 (0.72, 0.82)	0.84 (0.78, 0.90)	0.91 (0.83, 0.99)	0.89 (0.81, 0.98)
Walking activity								
No	1.00 (reference)	1.00 (reference)	1.00 (reference)	1.00 (reference)	1.00 (reference)	1.00 (reference)	1.00 (reference)	1.00 (reference)
Yes	1.02 (0.96, 1.09)	1.00 (0.94, 1.07)	1.19 (1.08, 1.31)	1.20 (1.08, 1.33)	1.16 (1.09, 1.22)	1.15 (1.08, 1.22)	1.09 (1.00, 1.20)	1.06 (0.96, 1.18)
Perceived health status								
Bad	1.00 (reference)	1.00 (reference)	1.00 (reference)	1.00 (reference)	1.00 (reference)	1.00 (reference)	1.00 (reference)	1.00 (reference)
Average	0.73 (0.66, 0.80)	0.91 (0.82, 100)	0.93 (0.83, 1.05)	0.94 (0.83, 1.07)	0.72 (0.67, 0.77)	0.91 (0.84, 0.98)	0.96 (0.86, 1.05)	0.99 (0.89, 1.11)
Good	0.63 (0.58, 0.69)	0.84 (0.75, 0.93)	0.68 (0.61, 0.77)	0.70 (0.61, 0.81)	0.54 (0.49, 0.58)	0.77 (0.70, 0.84)	0.67 (0.59, 0.76)	0.77 (0.67, 0.90)
Health screening								
No	1.00 (reference)	1.00 (reference)	1.00 (reference)	1.00 (reference)	1.00 (reference)	1.00 (reference)	1.00 (reference)	1.00 (reference)
Yes	2.39 (2.20, 2.61)	2.29 (2.09, 2.51)	3.09 (2.79, 3.43)	2.68 (2.39, 3.00)	2.10 (1.94, 2.27)	2.07 (1.91, 2.25)	2.60 (2.38, 2.86)	2.36 (2.13, 2.60)
Public health centers use								
No	1.00 (reference)	1.00 (reference)	1.00 (reference)	1.00 (reference)	1.00 (reference)	1.00 (reference)	1.00 (reference)	1.00 (reference)
Yes	2.61 (2.43, 2.80)	2.36 (2.19, 2.54)	4.52 (4.06, 5.02)	4.31 (3.85, 4.82)	1.89 (1.78, 2.00)	1.77 (1.66, 1.88)	3.80 (3.46, 4.18)	3.66 (3.31, 4.05)

OR, odds ratio; CI, confidence interval.

1 Adjusted OR and 95% CI for influenza vaccination according to socio-demographic characteristics, health behaviors, chronic diseases, use of healthcare services by age group.

**Table 5.** Factors associated with Influenza vaccination in the study population by chronic disease

Variable	Men				Women			
	50-64 yr		≥65 yr		50-64 yr		≥65 yr	
	Crude OR (95% CI)	Adjusted OR (95% CI)^1^	Crude OR (95% CI)	Adjusted OR (95% CI)^1^	Crude OR (95% CI)	Adjusted OR (95% CI)^1^	Crude OR (95% CI)	Adjusted OR (95% CI)^1^
Hypertension^2^								
No	1.00 (reference)	1.00 (reference)	1.00 (reference)	1.00 (reference)	1.00 (reference)	1.00 (reference)	1.00 (reference)	1.00 (reference)
Yes	1.53 (1.43, 1.63)	1.27 (1.19, 1.37)	1.59 (1.44, 1.75)	1.34 (1.20, 1.49)	1.73 (1.62, 1.84)	1.39 (1.30, 1.49)	1.69 (1.54, 1.85)	1.55 (1.40, 1.71)
Diabetes								
No	1.00 (reference)	1.00 (reference)	1.00 (reference)	1.00 (reference)	1.00 (reference)	1.00 (reference)	1.00 (reference)	1.00 (reference)
Yes	1.63 (1.50, 1.77)	1.41 (1.28, 1.55)	1.29 (1.14, 1.45)	1.17 (1.02, 1.33)	2.02 (1.83, 2.22)	1.51 (1.35, 1.68)	1.37 (1.22, 1.53)	1.27 (1.12, 1.43)
Chronic cardiovascular disease								
No	1.00 (reference)	1.00 (reference)	1.00 (reference)	1.00 (reference)	1.00 (reference)	1.00 (reference)	1.00 (reference)	1.00 (reference)
Yes	1.54 (1.32, 1.81)	1.11 (0.94, 1.32)	1.55 (1.29, 1.87)	1.31 (1.07, 1.60)	1.89 (1.56, 2.30)	1.31 (1.05, 1.64)	1.20 (1.00, 1.44)	1.09 (0.90, 1.32)
Stroke								
No	1.00 (reference)	1.00 (reference)	1.00 (reference)	1.00 (reference)	1.00 (reference)	1.00 (reference)	1.00 (reference)	1.00 (reference)
Yes	1.67 (1.36, 2.04)	1.22 (0.97, 1.53)	1.17 (0.98, 1.40)	1.04 (0.85, 1.27)	1.62 (1.24, 2.12)	1.18 (0.90, 1.55)	1.09 (0.91, 1.32)	0.98 (0.80, 1.19)

OR, odds ratio; CI, confidence interval.

1 Adjusted OR and 95% CI for influenza vaccination according to sociodemographic characteristics, health behaviors, chronic diseases, use of healthcare services by age group.

2 Excluded from the high-risk group.

**Table 6.** Coverage rates of influenza vaccinations by region in the study population, by chronic diseases

Variables	50-64 yr				≥65 yr			
	No high-risk conditions		≥1 high-risk conditions^1^		No high-risk conditions		≥1 high-risk conditions^1^	
	Total	Vaccinated	Total	Vaccinated	Total	Vaccinated	Total	Vaccinated
Nationwide	56,449	22,397 (35.6)	10,026	5,113 (47.2)	44,535	38,760 (85.2)	19,598	17,502 (88.5)
Seoul Metropolitan City	5,429	1,840 (32.6)	801	366 (42.1)	3,073	2,590 (84.4)	1,405	1,257(89.4)
Busan Metropolitan City	3,736	1,306 (33.3)	657	326 (48.3)	2,283	1,880 (82.0)	1,166	1,007 (85.8)
Daegu Metropolitan City	1,735	638 (34.9)	288	145 (49.5)	1,170	985 (83.1)	507	453 (89.0)
Incheon Metropolitan City	2,148	937 (38.2)	400	223 (54.3)	1,449	1,255 (83.0)	700	624(86.8)
Gwangju Metropolitan City	1,062	374 (33.9)	148	70 (47.4)	595	494 (81.4)	297	255 (86.8)
Daejeon Metropolitan City	1,088	381 (34.6)	175	86 (48.0)	532	448 (82.8)	287	255 (87.5)
Ulsan Metropolitan City	1,172	488 (40.4)	176	86 (47.4)	461	394 (85.1)	222	195 (87.1)
Gyeonggi-do	9,779	3,561 (35.0)	1,595	754 (46.2)	4,931	4,238 (86.0)	2,379	2,136 (89.7)
Gangwon-do	4,348	1,785 (37.8)	895	445 (46.1)	3,325	2,880 (86.1)	1,553	1,385 (87.2)
Chungcheongbuk-do	3,221	1,388 (39.1)	661	370 (49.7)	2,640	2,355 (89.5)	1,228	1,136 (91.5)
Chungcheongnam-do	3,361	1,487 (40.0)	694	369 (47.2)	3,183	2,839 (88.9)	1,370	1,233 (89.2)
Jeollabuk-do	2,928	1,409 (41.1)	549	326 (52.2)	3,618	3,268 (88.0)	1,525	1,398 (90.6)
Jeollanam-do	4,585	2,219 (44.1)	971	564 (52.5)	6,006	5,385 (87.5)	2,530	2,279 (89.1)
Gyeongsangbuk-do	5,756	2,279 (35.1)	990	495 (48.6)	5,794	5,101 (86.6)	2,368	2,087 (88.2)
Gyeongsangnam-do	4,604	1,829 (35.5)	811	395 (45.9)	4,233	3,579 (83.4)	1,607	1,402 (84.2)
Jeju Island	1,321	410 (31.4)	177	73 (36.3)	1,101	950 (84.7)	390	341 (86.9)
Sejong Metropolitan Autonomous City	176	66 (34.2)	38	20 (48.0)	141	119 (82.3)	64	59 (92.5)

Values are presented as number or number (weighted %).

1 Adults who had at least one of the following: diabetes, myocardial infarction or angina, stroke.

## SUPPLEMENTARY MATERIALS

Supplementary Material: Korean version is available at http://www.e-epih.org/.
