# Supplementary material for: The coverage rates for influenza vaccination and related factors in Korean adults aged 50 and older with chronic disease: based on 2016 Community Health Survey data
Source: Epidemiol Health. 2018 Jul 24;40:e2018034. doi: 10.4178/epih.e2018034 (PMC6232656; doi:10.4178/epih.e2018034)
Supplement: Supplementary file 1 [file epih-40-e2018034-supplementary.pdf]

Volume: 40, Article ID: e2018034

https://doi.org/10.4178/epih.e2018034

The estimated proportion for influenza vaccination and related factors in Korean adults aged 50 and older with chronic disease: based on 2016 Community Health Survey data

Kyeong Hyang Byeon 1,2, Jai Yong Kim 2, Bo Young Choi 3, Bo Youl Choi 2

1Department of Public Health, Graduate School, Hanyang University, Seoul, Korea; 2Department of Preventive Medicine, Hanyang University College of Medicine, Seoul, Korea; 3Department of Public Health & Medical Administration, Dongyang University, Yeongju, Korea

Corresponding Author: Bo Youl Choi

Department of Preventive Medicine, Hanyang University College of Medicine, 222 Wangsimni-ro, Seongdong-gu, Seoul, 04763, Republic of Korea

E-mail: bychoi@ehanyang.ac.kr

Volume: 40, Article ID: e2018034

https://doi.org/10.4178/epih.e2018034

Abstract

Objectives

This study aim to identify the estimated proportion for influenza vaccination and related factors depending on chronic disease in Korean adults aged 50 and older.

Methods

The 2016 Community Health Survey(CHS) was used for analysis. Chi-square test was performed to investigate the estimated proportion for influenza vaccination depending on chronic disease, and a multiple logistic regression analysis was used to identify the factors associated with influenza vaccination, by chronic disease.

Results

In Men with ≥1 chronic disease, 39.8% of 50-64 years of age, and 86.8% of elderly (over 65 years of age) received influenza vaccination. In women with ≥1 chronic disease, 58.7% of 50-64 years of age, and 89.9% of elderly (over 65 years of age) received influenza vaccination ($p<0.0001$).

The chronic diseases associated with Influenza vaccination were hypertension (odds ratio [OR=1.27, 95% CI: 1.19-1.37), diabetes (OR=1.41, 95% CI: 1.28-1.55) in men aged 50-64, hypertension(OR=1.34, 95% CI: 1.20-1.49), diabetes(OR=1.17, 95% CI: 1.02-1.33), chronic cardiovascular disease(OR=1.31, 95% CI: 1.07-1.60) in elderly (over 65 years of age). In women aged 50-64, hypertension (OR=1.39, 95% CI: 1.30-1.49), diabetes (OR=1.51, 95% CI: 1.35-1.68), chronic cardiovascular disease (OR=1.31, 95% CI: 1.05-1.64), and hypertension (OR=1.55, 95% CI: 1.40-1.71), diabetes (OR=1.27, 95% CI: 1.12-1.43) in elderly (over 65 years of age).

Volume: 40, Article ID: e2018034

https://doi.org/10.4178/epih.e2018034

Conclusions

Populations in aged 50-64 are recommendation subject for vaccination or classified as high risk group in case with chronic disease. Though subject over 60 years old is age close to the elderly, the estimated proportion for vaccination was low. It is necessary to devise strategies to raise the estimated proportion for vaccination.

Key words : Influenza vaccination, Chronic disease, High risk group, Community Health Survey.

Volume: 40, Article ID: e2018034

https://doi.org/10.4178/epih.e2018034

Ⅰ. 서론

인플루엔자는 인플루엔자 바이러스 감염에 의한 급성 호흡기 질환으로 노인 및 만성질환자는 중증 인플루엔자에 이환 되거나 합병증 발생 가능성이 높아 고위험군으로 정의되고 있다. 고위험군 환자에서는 폐렴과 같은 합병증의 진행 위험이 높다. 일부 환자는 중증 합병증으로 인해 입원치료 및 중환자실 집중치료가 필요하며 사망하는 경우도 있다 [1].

국내에서 2015년 국민건강보험공단 자료에 의하면, 인플루엔자(J10)로 65세 이상 입원 진료를 본 환자는 5,293명, 50-64세 성인은 5,453명, 30-49세는 6,412명으로 연령이 낮을수록 인플루엔자로 인한 입원 환자수가 증가하는 것으로 나타났다 [2]. 전국 주민등록 인구수 대비 인구 10만명당 입원환자 수는 65세 이상은 78.1명, 50-64세는 48.7명, 30-49세는 38.8명으로 확인되었다 [3]. 인플루엔자는 예방접종으로 예방 가능하므로 유행 전 예방접종이 필수적이며, 유행 시 지역사회의 공중보건학적 문제를 초래할 수 있다 [4].

예방접종은 인플루엔자를 예방할 수 있는 가장 비용 효과적인 방법이고, 예방접종의 효과는 피접종자의 연령, 기저질환 등에 따라 다양하다. 건강한 성인에서 예방접종의 효과는 70~90%, 노인에서는 인플루엔자로 인한 입원 예방에는 50~60%, 사망을 예방하는데 80% 정도 효과가 있다고 나타났다 [5]. 만성질환자의 경우 인플루엔자 유사질환, 폐렴, 병원입원율, 사망률을 감소시킨다 [6]. 국내에서는 인플루엔자 유행주의보 발령 이전부터 65세 이상을 포함한 우선접종 권장대상자들에게 예방접종 할 것을 지속적으로 안내하고 있으며, 인플루엔자 유행 중이라도 미접종자의 경우 예방접종을 권장하고 있다 [7, 8].

미국에서 65세 이상 예방접종률은 63.4%, 50-60세는 43.6%로 나타났고, 국내 65세 이상은 81.7%, 50-64세는 28.4% 확인되었다 [5, 9-11]. 국내 50-64세 연령의 예방접종률 수치는 인플루엔자 우선접종 권장대상자임을 고려한다면 매우 낮다고 평가할 수 있다.

미국 Healthy People 2020에서는 고위험군 환자로 분류된 65세 이상 노인과 만성질환을 가진

Volume: 40, Article ID: e2018034

https://doi.org/10.4178/epih.e2018034

사람들의 인플루엔자 예방접종률을 90% 달성하는 것을 목표로 하고, 국내에서는 65세 이상 노인의 예방접종률을 82%까지 달성하는 것을 목표로 한다. 그러나 고위험군으로 정의된 만성질환자의 경우 예방접종 달성 목표가 명시되지 않았으며, 선행연구를 통해 만성질환이 동반되었을 경우 예방접종률이 낮은 것을 확인할 수 있었다 [12, 13].

본 연구는 2016년 50-64세 연령에서 만성질환을 동반할 경우 인플루엔자 예방접종률을 확인하고, 실제 예방접종에 영향을 주는 요인을 파악하여 50-64세 연령에서 예방접종률을 높이기 위한 기초자료로 활용하고자 수행하였다.

Ⅱ. 연구방법

1. 연구자료 및 대상

2016년 지역사회건강조사 원시자료를 이용하여 인플루엔자 예방접종 설문문항에 응답한 50세 이상 남자 56,917명, 여자 73,693명을 최종 연구대상자로 선정하였다 [14].

2. 변수 선정 및 정의

본 연구의 종속변수는 인플루엔자 예방접종 변수로서 예방접종을 한 사람을 예로, 하지 않은 사람을 아니오로 정의하였다.

독립변수의 사회경제적 요인은 성별, 연령, 시도, 가구수입, 교육수준, 직업분류, 결혼상태 변수를 사용하였고, 건강행태는 흡연, 음주, 걷기활동, 주관적 건강수준이며, 만성질환은 고혈압, 당뇨병, 심근경색 또는 협심증, 뇌졸중 진단여부로 정의하였다. 고혈압을 제외한 만성질환들은 중증 인플루엔자 이환 또는 합병증 발생 위험이 높아 인플루엔자 예방접종 고위험군(High risk group) 대상자로 구분한다 [1]. 의료서비스 이용은 건강검진과 보건기관 이용 변수를 사용하였다.

3. 분석방법

Volume: 40, Article ID: e2018034

https://doi.org/10.4178/epih.e2018034

지역사회건강조사 자료는 복합표본설계 자료로서 각 분석 과정에 가중치, 층, 집락을 포함하고, SURVEY 프로시저를 사용하여 분석하였다.

대상자의 사회경제적 요인, 건강행태 요인, 만성질환 여부, 의료서비스 이용 요인과 인플루엔자 예방접종과의 유의한 차이를 보기 위해 카이제곱검정을 시행하였다. 또한 50세 이상 대상자의 인플루엔자 예방접종에 영향을 미치는 요인을 확인하기 위해 다중 로지스틱 회귀분석을 실시하여 오즈비와 95% 신뢰구간을 산출하였다. 지역은 시도 및 대도시, 중소도시, 군지역으로 구분하여 50세 이상 대상자의 1개 이상 만성질환 여부에 따른 인플루엔자 예방접종률을 확인하였다.

자료 분석은 SAS 9.4 (SAS Institute Inc. Cary. NC, USA)를 사용하여 수행하였다.

Ⅲ. 연구결과

1. 50세 이상에서 연령그룹에 따른 인플루엔자 예방접종률

연령그룹에 따른 예방접종률을 보면 50-54세에서 남자는 23.2%, 여자는 31.3%, 65-69세에서 남자는 76.9%, 여자는 83.3%로 확인되었다(Table 1). 50-64세 연령대에서는 남녀 60-64세에서 높은 예방접종률을 보였고, 65세 이상 연령대에서는 남자 80-84세에서 90.2%, 여자 75-79세에서 91.1%로 높은 예방접종률을 볼 수 있었다.

2. 50세 이상에서 건강 관련 요인과 인플루엔자 예방접종률의 특성

50-64세, 65세 이상으로 구분하여 예방접종률을 보면, 50-64세 남자는 30.5%, 여자는 43.8%, 65세 이상 남자는 84.3%, 여자는 87.8%이며, 여자에서 예방접종률이 더 높은 것으로 확인되었다(Table 2). 가구소득은 하에서 50-64세 남자는 35.9%, 여자는 52.1%, 65세 이상 남자는 85.1%로 예방접종률이 가장 높았으며, 여자에서 가구소득에 따른 예방접종률은 통계적 차이가 없었다. 교육 수준은 50-64세 남녀, 65세 이상 여자에서 교육수준이 높을수록 낮은

https://doi.org/10.4178/epih.e2018034

예방접종률을 보였으며, 65세 이상 남자의 경우 유의한 차이가 없었다. 직업분류는 연령그룹 및 남녀 모두에서 무직일 경우 예방접종률이 높았다. 결혼상태에서 남자는 유배우자와 동거일 경우 50-64세 31.2%, 65세 이상 85.1%로 높은 예방접종률을 보였으며, 여자는 50-64세에서는 기타(이혼, 사별, 별거)에서 46.3%, 65세 이상에서는 미혼에서 90.2%의 가장 높은 예방접종률을 보였다. 남자는 이전 흡연자에서, 여자는 비흡연에서 높은 예방접종률을 보였으며, 음주여부에서는 50-64세 남녀 모두, 65세 이상 여자에서 비음주자 일 경우 88.3%로 예방접종률이 높게 나타났다. 50-64세 여자, 65세 이상 남녀 모두 걷기활동을 하는 군에서 높은 예방접종률을 보였다. 주관적 건강수준에서는 모든 그룹에서 건강수준이 나쁠수록 높은 예방접종률을 보였고, 남녀 모두 건강검진과 보건소 이용을 할 경우 예방접종률이 높게 나타났다.

3. 50세 이상에서 만성질환 여부와 인플루엔자 예방접종률의 특성

남녀 모두 고혈압, 당뇨병, 만성 심혈관 질환, 뇌졸중을 동반할 경우 예방접종률이 높았으며, 뇌졸중에서는 남녀 50-64세 그룹에서만 유병여부에 따른 예방접종률과의 유의한 차이를 보였다(Table 3). 1개 이상 만성질환을 동반할 경우 예방접종률을 보면, 50-64세에서 남자는 39.8%, 여자는 58.7%, 65세 이상에서 남자는 86.8%, 여자는 89.9%로 확인되었고, 만성질환을 동반할 경우 높은 예방접종률을 보였다. 만성질환의 개수에 따른 예방접종률을 보면, 50-64세에서 남자는 만성질환이 1개 동반 될 경우 39.3%, 2개는 43.1%, 3개는 53.8%, 여자는 1개 동반될 경우 58.2%, 2개는 65.3%, 3개는 88.7%로 나타났다. 65세 이상에서 남자는 만성질환이 1개 동반 될 경우 86.3%, 2개는 89.0%, 3개는 89.9%, 여자는 만성질환이 1개 또는 2개 동반 될 경우 모두 90.0%, 3개는 83.6%로 확인되어, 만성질환 동반 개수가 증가할수록 예방접종률이 높게 나타났다.

4. 50세 이상에서 인플루엔자 예방접종에 영향을 미치는 요인

인플루엔자 예방접종을 할 요인을 남자에서 보면, 50-64세에서는 가구소득이 중상일 경우 예방접종을 할 확률이 19.0% 낮았으며(CI: 0.70-0.92), 교육수준이 중졸 이하에 비해 고졸 일

Volume: 40, Article ID: e2018034

https://doi.org/10.4178/epih.e2018034

경우 29.0%(CI: 0.65-0.77), 대졸 이상 일 경우 43.0%(CI: 0.52-0.63) 예방접종을 할 확률이 낮게 나타났다(Table 4). 직업분류는 50-64세에서 사무직에 비해 육체노동일 경우 예방접종을 할 확률이 14.0% 낮았으며(CI: 0.78-0.94), 65세 이상에서 무직일 경우 예방접종의 오즈비가 1.41배 높았다(CI: 1.13-1.77). 50-64세에서 미혼자에 비해 결혼할 경우 유배우자가 있을 때 예방접종의 오즈비가 1.23배 높았고(CI: 1.02-1.47), 65세 이상에서는 유배우자가 있을 경우 3.49배(CI: 2.01-6.05), 기타는 2.57배(CI: 1.47-4.50) 높았다. 50-64세에서 현재 흡연자에 비해 과거 흡연자 일 경우 예방접종의 오즈비가 1.48배(CI: 1.38-1.60), 비흡연자는 1.56배(CI: 1.42-1.72) 높았고, 65세 이상에서 과거 흡연자 일 경우 1.61배(CI: 1.43-1.81), 비흡연자는 1.57배(CI: 1.34-1.82) 높았다. 50-64에서 음주할 경우 예방접종을 할 확률이 19.0% 낮았으며(CI: 0.71-0.92), 65세 이상에서 걷기활동을 할 경우 예방접종의 오즈비가 1.20배(CI: 1.08-1.33) 높았다. 50-64에서 주관적 건강상태가 나쁨에 비해 좋을 경우 예방접종을 할 확률이 16.0% 낮았으며(CI: 0.75-0.93), 65세 이상에서 좋을 경우 예방접종 할 확률이 30.0% 낮았다(CI: 0.61-0.81). 50-64세에서 건강검진을 할 경우 예방접종의 오즈비가 2.29배(CI: 2.09-2.51), 65세 이상에서 2.68배(CI: 2.39-3.00) 높았다. 50-64세에서 보건소 이용을 할 경우 예방접종의 오즈비가 2.36배(CI: 2.19-2.54), 65세 이상은 4.31배(CI: 3.85-4.82) 높았다.

여자에서 보면, 50-64세에서 가구소득이 중상일 경우 예방접종을 할 확률이 14.0% 낮았으며(CI: 0.77-0.95), 65세 이상에서 상일 경우 예방접종의 오즈비가 1.40배(CI: 1.14-1.70) 높았다. 교육수준은 50-64세에 중졸 이하에 비해 고졸 일 경우 예방접종을 할 확률이 34.0%(CI: 0.62-0.71), 대졸 이상일 경우 47.0%(CI: 0.48-0.58) 낮았다. 50-64세에서 직업분류가 육체노동일 경우 예방접종을 할 확률이 13.0% 낮았으며(CI: 0.78-0.94), 무직일 경우 예방접종의 오즈비가 1.17배(CI: 1.04-1.31) 높았고, 65세 이상에서도 무직일 경우 1.70배(CI: 1.12-2.60) 높았다. 50-64세에서 현재 흡연자에 비해 과거 흡연자일 경우 예방접종의 오즈비가 1.31배(CI: 1.01-1.70), 비흡연자는 1.55배(CI: 1.31-1.83) 높았으며, 65세 이상에서 과거 흡연자는 1.61배(CI: 1.12-2.32), 비흡연자는 1.73배(CI: 1.29-2.33) 높았다. 음주를 할 경우 50-64세에서 예방접종을 할

Volume: 40, Article ID: e2018034

https://doi.org/10.4178/epih.e2018034

확률이 16.0% 낮았으며(CI: 0.78-0.90), 65세 이상에서도 예방접종을 할 확률이 11.0% 낮았다(CI: 0.81-0.98). 50-64세에서 걷기활동을 할 경우 예방접종의 오즈비가 1.15배(CI: 1.08-1.22) 높았으며, 주관적 건강상태는 50-64세에서 나쁨에 비해 보통일 경우 예방접종 할 확률이 9.0%(CI: 0.84-0.98), 좋을 경우 23.0%(CI: 0.70-0.84) 낮았으며, 65세 이상에서 좋을 경우 예방접종을 할 확률이 23.0% 낮았다(CI: 0.67-0.90). 50-64세에서 건강검진을 할 경우 예방접종의 오즈비가 2.07배(CI: 1.91-2.25), 65세 이상에서 2.36배(CI: 2.13-2.60) 높았다. 보건소 이용을 할 경우 50-64세에서 예방접종의 오즈비가 1.77배(CI: 1.66-1.88), 65세 이상에서 3.66배(CI: 3.31-4.05) 높았다.

50세 이상 대상자에서 인플루엔자 예방접종에 영향을 미치는 만성질환 관련 요인은 Table 5와 같다. 50-64세 남자에서 고혈압이 있을 경우 예방접종의 오즈비가 1.27배(CI: 1.19-1.37), 당뇨는 1.41배(CI: 1.28-1.55) 높았다. 65세 이상에서 고혈압이 있을 경우 예방접종의 오즈비가 1.34배(CI: 1.20-1.49), 당뇨는 1.17배(CI: 1.02-1.33), 만성 심혈관질환은 1.31배(CI: 1.07-1.60) 높았다. 50-64세 여자에서 고혈압이 있을 경우 예방접종의 오즈비가 1.39배(CI: 1.30-1.49), 당뇨는 1.51배(CI: 1.35-1.68), 만성 심혈관 질환은 1.31배(CI: 1.05-1.64) 높았다. 65세 이상에서 고혈압이 있을 경우 예방접종의 오즈비가 1.55배(CI: 1.40-1.71), 당뇨는 1.27배(CI: 1.12-1.43) 높았다.

5. 지역별로 50세 이상에서 1개 이상 만성질환 여부에 따른 인플루엔자 예방접종률

시도에 따른 인플루엔자 예방접종률은 50-64세 연령에서는 전라남도가 44.1%로 높았고, 제주도가 31.4%로 가장 낮았다(Table 6). 1개 이상 만성질환을 가질 경우 인천광역시가 54.3%로 높았으며, 제주도가 36.3%로 가장 낮았다. 65세에서는 충청북도가 89.5%로 높았고, 광주광역시가 81.4%로 최저를 보였으며, 1개 이상 만성질환을 가질 경우 세종특별자치시가 92.5%, 충청북도가 91.5%로 예방접종률이 높았고, 경상남도가 84.2%로 가장 낮을 접종률을 보였다.

Volume: 40, Article ID: e2018034

https://doi.org/10.4178/epih.e2018034

대도시, 중소도시, 군지역의 65세 이상에서 2015년 예방접종률 80%의 목표치와 비교하여, 목표 달성을 하지 못한 지역의 수를 보면, 만성질환이 없는 경우 대도시에서는 16개 지역, 중소도시는 6개 지역, 군지역은 1개 지역이 80%를 달성하지 못하였고, 1개 이상 만성질환이 있는 경우 대도시에서 5개 지역, 중소도시는 4개 지역, 군지역은 없는 것으로 확인되었다(Fig 1)(Fig 2).

Ⅳ. 고찰

본 연구는 2016년 지역사회건강조사 자료를 이용하여 최근 1년 동안 인플루엔자 예방접종 여부에 응답한 50세 이상을 대상으로 만성질환을 동반할 경우, 지역별로 구분하여 1개 이상 만성질환을 동반할 경우 인플루엔자 예방접종률과 관련 요인들을 확인하였다.

국내에서는 2015년 이후 국가 예방접종 사업을 민간의료기관까지 확대하면서 65세 이상 노인의 인플루엔자 예방접종률을 80% 이상 달성하는 것을 목표로 했다 [15]. 65세 이상 대상자는 국가 예방접종 지원사업으로 인해 무료 접종을 실시하고 있어 높은 예방접종률을 보였고, 80% 이상 예방접종률을 달성하였다. 단, 65-69세 남자에서는 76.9%로 확인되었다. 반면 50-64세 인구는 2003년 이후 예방접종 우선접종 권장대상자에 추가되었지만 본 연구를 통해 남자는 30.5%, 여자는 43.8%로 예방접종률이 낮은 것을 확인함으로써 예방접종률을 올릴 수 있는 정책적 대안과 지역사회의 예방접종에 대한 홍보가 필요할 것으로 생각한다.

국내에서는 인플루엔자가 유행하기 전 우선접종 권장대상자들에게 예방접종을 권고하고 있다. 의료인, 임신부, 50-64세 인구, 65세 이상 노인, 만성폐질환자, 만성심장질환자, 만성간질환자, 만성신질환자, 신경-근육 질환, 혈액-종양 질환, 당뇨환자 등이 권장대상자에 포함되며, 이들 중 만성질환자는 고위험군으로 구분된다.

본 연구는 50세 이상 만성질환이 없는 사람들에 비해 만성질환을 동반할 경우 예방접종률이

Volume: 40, Article ID: e2018034

https://doi.org/10.4178/epih.e2018034

높은 것을 확인할 수 있었고, 만성질환의 개수가 1개 이상일 경우, 만성질환의 개수가 증가할 경우 높은 예방접종률을 보인 것은 국외 선행연구와 동일한 결과로 확인되었다 [16, 17]. 또한 고위험군에 해당되는 50-64세 연령에서 만성질환을 동반할 경우, 65세 이상 또는 만성질환을 동반할 경우 중증 인플루엔자에 이환 되거나 합병증 발생 가능성이 높음에도 불구하고 50-64세 연령의 만성질환자는 예방접종률이 낮은 것을 볼 수 있었다. 만성질환을 가지고 있는 사람은 인플루엔자 감염에 대한 감수성이 높아져 기저질환의 악화, 폐렴 발생, 다른 장기의 부전 등 합병증 발생 위험이 높아지며, 중증으로 진행하거나 사망에 이를 수 있기 때문에 인플루엔자 예방접종이 매우 중요하다. 50세 이상 남자는 심뇌혈관 질환을 동반할 경우 여자는 당뇨병을 동반할 경우 높은 예방접종률을 보였고, 남녀 모두 당뇨병 유병이 예방접종을 할 요인으로 확인되었다. 당뇨병 환자는 고혈당으로 감염에 대한 면역기능이 저하되어 폐렴, 패혈증과 같은 여러 심각한 합병증을 동반할 수 있기 때문에 예방접종률이 높은 것으로 생각한다.

건강 관련 요소에서는 남녀 모두 무직일 경우 예방접종률이 높았고, 65세 이상에서는 무직일 경우 예방접종 할 요인으로 나타났다. 선행연구에서는 너무 바빠서 예방접종을 받을 시간이 없는 것이 백신접종을 하지 못하는 이유인 것으로 확인되었고 [18, 19], 무직일 경우 예방접종을 받을 수 있는 시간적 여유가 있기 때문에 예방접종률이 높은 것으로 해석할 수 있다. 결혼상태에서는 남녀 모두 결혼을 했을 경우 예방접종률이 높았고, 남자에서는 결혼 상태가 예방접종을 할 요인으로 확인되었다. 국내 선행연구에서 배우자와 함께 생활 할 경우, 가족과 함께 생활 할 경우 높은 예방접종률을 보였으며 사회관계망을 나타내는 소셜 네트워크와 가족의 예방접종 권고는 건강 정보에 긍정적인 영향을 주는 것으로 생각된다 [6, 20]. 건강에 나쁜 생활습관인 흡연과 음주를 할 경우 65세 이상 남자 음주자를 제외하고는 남녀 예방접종률이 낮았고, 선행연구와 동일하게 건강한 생활습관을 가진 사람에서 건강을 보호하는 행동 및 건강을 위한 예방적 진료를 더 추구할 것이며 인플루엔자 백신 접종에도 관심이 높은 것으로 생각된다 [13]. 건강검진과 보건소 이용을 할 경우 남녀 모두 높은 예방접종률을 보였고, 예방접종을 할 요인으로 나타났다. 의료서비스 이용 요인은 선행연구에서 진료 시 의료인들과의 접촉이

Volume: 40, Article ID: e2018034

https://doi.org/10.4178/epih.e2018034

예방접종에 긍정적인 영향을 주는 것으로 확인되었고 [17, 21], 건강검진 및 보건소 이용을 하는 사람들은 조기에 질병을 예방하고 질병 및 건강증진 활동에 더 관심이 많기 때문에 예방접종률이 높은 것으로 생각된다.

지역에 따른 예방접종률을 보면 도보다는 광역시에 거주할 경우 높은 예방접종률을 보였고, 시에 거주할 경우 의료기관의 접근성이 좋기 때문에 예방접종률이 높은 것으로 생각된다. 또한 65세 이상에서는 무료 예방접종의 실시가 민간 의료기관까지 확대되어 시에 거주하는 사람들의 예방접종률을 높이는 것으로 보인다. 대도시, 중소도시, 군지역에서도 1개 이상 만성질환이 있을 경우 높은 예방접종률을 보였고, 군지역에서는 65세 이상에서 1개 이상 만성질환이 있을 경우 2015년 예방접종률 80% 목표치를 전 지역에서 달성하였다. 군지역에서 높은 예방접종률을 보인 것은 기존의 연구와 일치했으며 [22], 농촌지역에서는 도시지역보다 예방접종에 대한 홍보나 교육이 지역사회 보건소를 통해 주민들에게 이루어져 도시보다는 파급효과가 클 것으로 보인다 [23].

본 연구의 제한점은 첫째 인플루엔자 예방접종 여부가 설문지 형태의 자기 기입식 작성 방법이므로 실제 예방접종률과 차이가 날 수 있으며 예방접종률이 과대추정 될 수 있다. 둘째 지역사회건강조사는 지난 1년 동안의 인플루엔자 예방접종 여부를 묻는 문항에서 대상자의 회상바이어스가 발생 할 수 있는 가능성을 배제 할 수 없다. 셋째 만성질환의 진단 여부는 설문지 문항의 제한으로 인플루엔자 합병증 위험이 높은 여러 만성질환들의 예방접종률을 확인할 수 없었다. 넷째 노인 및 만성질환자의 경우 의료진의 추천, 이전의 예방접종 여부가 인플루엔자 예방접종을 할 강력한 요인임에도 해당변수가 없어 파악할 수 없었다 [24].

그럼에도 본 연구에서는 대상자의 지역적 특성을 고려한 조사 자료를 가지고 시도 및 대도시, 중소도시, 군지역으로 분류하여 50-64세, 65세 이상 연령에서 만성질환 동반 여부에 따른 예방접종률을 확인하고, 예방접종에 영향을 주는 요인을 확인하였다. 50-64세 연령에서 1개 이상 만성질환을 동반할 경우 대도시, 중소도시에 거주하는 것보다 군지역에 거주할 경우 높은

예방접종률을 확인함으로써 대도시 및 중소도시에 거주하는 50-64세 연령에 대해 예방접종률을 올릴 수 있는 적극적인 예방접종 홍보가 필요할 것으로 본다. 또한, 50-64세 만성질환자가 의료기관을 방문하여 진료를 받을 경우 의료진의 예방접종이 권유되어야 할 것으로 생각한다.

REFERENCES

1. Baek JH, Seo YB, Choi WS, Kee SY, Jeong HW, Lee HY, et al. Guideline on the prevention and control of seasonal influenza in healthcare setting. Korean J Intern Med 2014;29:265-280.
2. NHISS. National Health Insurance Sharing Service [cited 2017 Dec 28]. Available from: https://nhiss.nhis.or.kr/bd/ad/bdada033cv.do.
3. KOSIS KSIS. Population resident registration (2011 ~) by administrative area (Eup, Myeon-dong) / 5 years old [cited 2018 11 May]. Available from: http://kosis.kr/statHtml/statHtml.do?orgId=101&tblId=DT_1B04005N&conn_path=I3.
4. Casanova L, Gobin N, Villani P, Verger P. Bias in the measure of the effectiveness of seasonal influenza vaccination among diabetics. Prim Care Diabetes 2016;10:398-406.
5. KCDC. 2017-2018 seasonal Influenza National Immunization Program Support project Management Guidelines for medical institutes [cited 201 Dec 15]. Available from: http://www.cdc.go.kr/CDC/notice/CdcKrTogether0302.jsp?menuIds=HOME001-MNU1154-MNU0005-MNU0088&cid=75943.
6. Kwon DS, Kim K, Park SM. Factors associated with influenza vaccination coverage among the elderly in South Korea: the Fourth Korean National Health and Nutrition Examination Survey (KNHANES IV). BMJ Open 2016;6:e012618.
7. Park YH, Kim BK, Jang WG, Chio BG, Lim SM, Kim EJ. A Study on the Supply and Demand of Influenza Vaccine in Korea. Journal of Soonchunhyang Medical Science 2009;15:35-44.
8. KCDC. Influenza vaccination [cited 2017 Dec 12]. Available from:

http://www.cdc.go.kr/CDC/contents/CdcKrContentView.jsp?cid=68455&menuIds=HOME001-MNU1132-MNU2430-MNU2432-MNU2464.

9. CDC. Flu Vaccination Coverage, United States, 2015-16 Influenza Season [cited 2017 Dec 12]. Available from: https://www.cdc.gov/flu/fluvaxview/coverage-1516estimates.htm.
10. KCDC. Korea health statistics 2015 [cited 2017 Dec 22]. Available from: https://knhanes.cdc.go.kr/knhanes/main.do.
11. Shin HN. status of influenza vaccination among Korean adults over 50years and its related factors [dissertation]. Seoul: Graduate School of Public Health Yonsei University; 2013.
12. Byeon G-R, Hur Y-I, Kang J-H, Park H-A, Kim K-W, Cho Y-G, et al. Influenza Vaccination Status in Korean Adult Population in Relation with Socioeconomic and Medical Factors. Korean J Health Promot 2016;16:20-31.
13. Lee KC, Han K, Kim JY, Nam GE, Han BD, Shin KE, et al. Socioeconomic status and other related factors of seasonal influenza vaccination in the South Korean adult population based on a nationwide cross-sectional study. PLoS ONE 2015;10 (2) (no pagination)
14. KCDC. Community Health Survey [cited 2018 Mar 7]. Available from: https://chs.cdc.go.kr/chs/index.do.
15. Lee HY YJ, Hong JI. Elderly Immunization Program against Influenza in Korea, 2015 [cited 2018 May 12]. Available from: http://www.cdc.go.kr/CDC/info/CdcKrInfo0301.jsp?menuIds=HOME001-MNU1154-MNU0005-MNU0037-MNU1380&cid=67630.
16. Annunziata K, Rak A, Del Buono H, DiBonaventura M, Krishnarajah G. Vaccination Rates among the General Adult Population and High-Risk Groups in the United States. PLoS ONE 2012;7 (11) (no pagination)
17. O'Halloran AC, Lu PJ, Williams WW, Bridges CB, Singleton JA. Influenza Vaccination Coverage Among People With High-Risk Conditions in the U.S. Am J Prev Med 2016;50:e15-26.

18. Kee SY, Lee JS, Cheong HJ, Chun BC, Song JY, Choi WS, et al. Influenza vaccine coverage rates and perceptions on vaccination in South Korea. J Infect 2007;55:273-281.
19. Kroneman M, van Essen GA, John Paget W. Influenza vaccination coverage and reasons to refrain among high-risk persons in four European countries. Vaccine 2006;24:622-628.
20. Cho HM, Kim JY, Hwang SE, Kim JC, Kim MY, Lee SH. Association between Living Arrangements and Influenza Vaccination Rates among Elderly South Korean People: The Fifth Korea National Health and Nutrition Examination Survey (KNHANES V-2). Korean J Fam Med 2015;36:186-190.
21. Wahid ST, Nag S, Bilous RW, Marshall SM, Robinson AC. Audit of influenza and pneumococcal vaccination uptake in diabetic patients attending secondary care in the Northern Region. Diabet Med 2001;18:599-603.
22. Ryu SY, Kim SH, Park HS, Park J. Influenza vaccination among adults 65 years or older: a 2009-2010 community health survey in the Honam region of Korea. Int J Environ Res Public Health 2011;8:4197-4206.
23. Park MB, Kim CB, Joo HS. Factors Influencing on Influenza Vaccination Coverage. J. of Contents Association 2013;13:300-311.
24. Yeung MP, Lam FL, Coker R. Factors associated with the uptake of seasonal influenza vaccination in adults: a systematic review. J Public Health (Oxf) 2016;38:746-753.
